# Preventing Breast Cancer-Related Lymphedema: Feasibility of Axillary Reverse Mapping Technique

**DOI:** 10.3390/jcm10235707

**Published:** 2021-12-06

**Authors:** Alexandra Caziuc, Diana Schlanger, Giorgiana Amarinei, Vlad Fagarasan, David Andras, George Calin Dindelegan

**Affiliations:** 1 1st Surgical Clinic, Surgical Department, University of Medicine and Pharmacy Cluj Napoca, 400006 Cluj Napoca, Romania; caziuc.alexandra@umfcluj.ro (A.C.); giorgianaamarinei@yahoo.com (G.A.); fagarasan.vlad@elearn.umfcluj.ro (V.F.); andras.david@umfcluj.ro (D.A.); george.dindelegan@umfcluj.ro (G.C.D.); 2 3rd Surgical Clinic, Surgical Department, University of Medicine and Pharmacy Cluj Napoca, 400162 Cluj Napoca, Romania

**Keywords:** breast cancer, lymphedema, axillary reverse mapping, lymph node, metastases

## Abstract

Introduction. Our study aimed to determine the feasibility of axillary reverse mapping (ARM) technique, the identification rate of ARM nodes and their metastatic involvement, as well as to identify the factors that influence the identification and metastatic involvement. Material and methods. In total, 30 breast cancer patients scheduled for axillary lymph node dissection were enrolled in our study. The lymphatic nodes that drain the arm were identified by injecting 1 mL of blue dye in the ipsilateral upper arm; then, the ARM nodes were resected along with the other lymph nodes and sent for histological evaluation. Results. Identification of ARM node was successful in 18 patients (60%) and 22.22% of the identified ARM lymph nodes had metastatic involvement. Patients with identified ARM nodes had a significant lower BMI and a statistically significant relationship between axillary lymph node status and ARM node metastases was proven. Most of ARM lymph nodes (96.3%) were found above the intercostobrachial nerve, under the axillary vein and lateral to the thoracodorsal bundle. Conclusions. The ARM procedure is easy to reproduce but might not be appropriate for patients with a high BMI. The rate of metastatic involvement of ARM nodes is significant and no factor can predict it, showing that the preservation of these nodes cannot be considered.

## 1. Introduction

Breast cancer-related lymphedema (BCRL) is a relatively common complication of breast cancer surgery. BCRL has detrimental effects on quality of life, alterations in arm function, or an increased risk for infections. Depending on the diagnostic criteria used, type of surgical treatment, and duration of follow up, recent studies estimated that 7% to 77% of breast cancer survivors eventually develop lymphedema [1]. Despite improvements in the management of the axilla (“less is more”), axillary lymph node dissection (ALND) remains a common surgical intervention for some patients with involved nodes or positive sentinel nodes. When ALND is combined with radiation therapy (RT), lymphedema rates are higher than with ALND alone (30%–50%) [1]. Although the use of sentinel node biopsy (SNB) has been shown to reduce the risk of lymphedema, recent studies cited an incidence of 4%–7%, which is still clinically significant [1,2]. Measures to reduce damage to the lymphatic drainage to the arm are therefore highly desirable.

Axillary reverse mapping (ARM) is a procedure that has been developed to reduce BCRL. This procedure is based upon the theory that the lymphatic drainage of the arm and the drainage of the breast take place on separate pathways in the axilla; these being considered, identifying and preserving the lymph path of the arm during surgical dissection, can reduce the postoperative lymphedema rate [3,4]. There are still questions regarding the feasibility of the technique, the oncological safety and the effectiveness in lymphedema prevention, the first two issues being discussed in detail in our study.

Our purpose was to determine the feasibility of the technique in a center with no previous experience with ARM procedure, to analyze the identification rate of ARM nodes and the factors that might influence it, to discuss the metastatic involvement of ARM nodes and the variables that might predict it and to identify the usual anatomical localization of the nodes that are involved in the lymphatic drainage of the arm.

## 2. Materials and Methods

### 2.1. Population Study

We performed a prospective study on 30 breast cancer patients scheduled for ALND. All procedures were conducted by the same surgical team in County Emergency Hospital Cluj-Napoca, First Surgical Clinic. The study protocol was approved by the Ethics Committee (312/05.02.2018).

The study included females, above 18 years old, diagnosed with invasive breast cancer and scheduled for axillary lymph node dissection (Table 1). Indications for ALND included clinically and biopsy proven lymph node metastases in the axilla or a history of axillary positive SNB.

Axillary recurrence or prior surgeries that could alter the lymphatic drainage of the arm were considered exclusion criteria. The included patients were informed about the protocol study and patient consent form was obtained for each participant.

### 2.2. Surgical Technique

**Figure 1:**
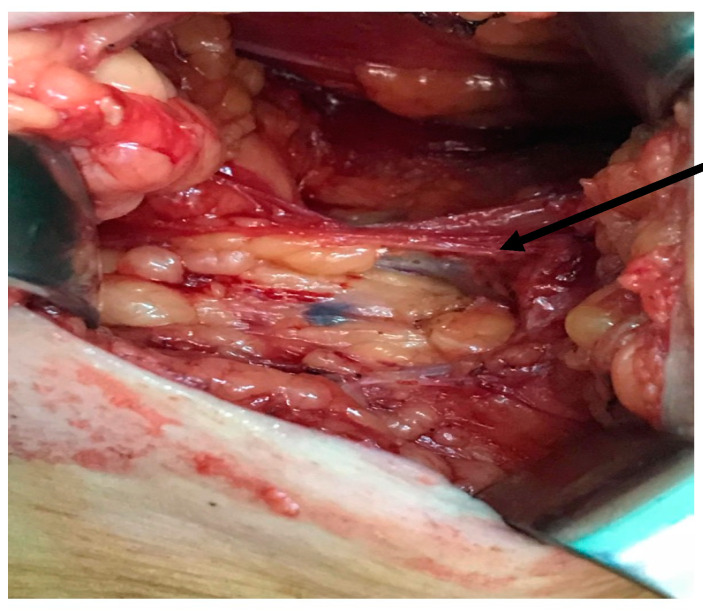
Axillary dissection—ARM node colored in blue.

Immediately after the patient was anesthetized, 1 mL of blue dye was injected intradermal and subcutaneously (0.5 mL intradermal and 0.5 mL subcutaneously) into the intermuscular groove of the upper inner arm on the side of the breast cancer. Then, the arm was elevated, and the site of the injection was massaged for 5 min. Next, the patient underwent breast surgery, followed by ALND, during which the ARM node (colored in blue) was identified (Figure 1), its anatomical location and the time between the injection of blue dye and identification were noted; after that it was resected with the other axillary lymph nodes. Resected ARM nodes were sent for histologic examination separately from the other nodes.

For a better systematization of the anatomical localization of ARM lymph nodes in the axilla, we used the classification described by Ikeda et al., in which the axilla is divided into 5 fields using surgical landmarks (the axillary vein, the thoracodorsal bundle, and the second intercostobrachial nerve) [5]. Field A is the area under the axillary vein, lateral to the thoracodorsal bundle, and superior to the second intercostobrachial nerve. Field B is the area localized medial to the thoracodorsal bundle, under the axillary vein and above the second intercostobrachial nerve. Field C and D are delimited superior by the second intercostobrachial nerve, field C being medial to the thoracodorsal bundle, while field D is lateral to it. Field E is the area above the axillary vein.

### 2.3. Studied Parameters

Information on age, body mass index (BMI), stage of disease, neoadjuvant treatment, tumor characteristics (tumor size, histological type, grade, HER2 expression, hormonal receptors, ki67 index), identification of ARM nodes, their number and location, axillary lymph node metastases and the presence of ARM metastases were collected for each participant (see Table 1).

### 2.4. Statistical Analysis

The statistical analysis was performed using IBM^®^ SPSS^®^ Statistics 26th version, USA, 2021 for Windows. Several statistic tests were used: T Student, Chi square, Mann-Whitney, depending on the type and distribution of the variables. A value of *p* < 0.05 was considered to be statistically significant, while a *p* value smaller than 0.01 was considered highly significant.

## 3. Results

### 3.1. Study Population

The clinical characteristics of the study participants are summarized in Table 1 of the 30 patients included in the study, 10% (3 patients) had SLB prior to ALND. The average age was 56.73 years (SD ± 12.56) and the average body mass index was 27.08 (SD ± 5.63). In total, 2 patients were underweight (BMI < 18.5), 11 patients were overweight (BMI between 25 and 30), and 9 patients were obese (BMI > 30).

### 3.2. Identification of ARM Nodes

Identification of ARM nodes was successful in 18 patients (60%). In 9 patients, 2 ARM nodes were found, the other 9 patients had 1 ARM node each, so a total of 27 ARM nodes were analyzed. In total, 70.37% (19 nodes) were localized in Field A, 25.93% (7 nodes) were localized in Field B, and 3.7% (1 node) were in Field D. There were no ARM nodes identified in Field C or E. Most ARM lymph nodes (26 nodes—96.3%) were identified above the intercostobrachial nerve (Field A and B). In total, 22.22% of the identified ARM lymph nodes had metastatic involvement. Out of the 18 patients in which the identification of ARM node was successful, 4 had metastasis in these nodes.

We conducted a comparison between the patients that had an ARM node identified and the patients in which no ARM node was found, in order to determine the possible factors that might influence the identification of ARM nodes; this comparison is detailed in Table 1. Patients with identified ARM nodes had a significantly lower BMI (*p* < 0.05). The other variables did not demonstrate significant differences between the two groups.

The mean time between injection and identification of ARM node was 23.03 min (SD ± 9.55), with no significant differences between the patients.

### 3.3. Metastatic Involvement of ARM Nodes

A correlation between the characteristics of the patient and the presence of metastatic involvement in ARM nodes was investigated. The only statistically significant correlation was with the histological lymph node status pN (*p* < 0.01—highly significant). The factors that might influence the metastatic involvement of ARM nodes were detailed in Table 2.

We analyzed the possible correlation between the metastatic status of ARM node and the anatomical localization of these nodes. The statistical analysis showed no significant relationship between these variables.

## 4. Discussion

Within our cohort, the descriptive analysis regarding tumor size, histology, intrinsic subtype of breast cancer is comparable with the data published by other authors in larger studies [6]. The ARM concept refers to drainage mapping of the arm with blue dye, in order to preserve the lymph channels and nodes, and consequently avoid BCRL. In our study, the detection rate of ARM nodes was 60%. In the studies already published, the identification rate varies between 46.6% reported by Kuusk et al. (2014) and 93.4% reported by Yue et al. (2015) [7,8]. The average identification rate for the ARM technique using blue dye was 78.4% [7]. Our reported rate, although lower than the average, is consistent with the results of other studies [8,9,10,11,12]. The detection rate of ARM nodes is influenced by several technical details: the time interval between the blue dye injection and the surgical intervention, the elevation and massage of the injection site, the quantity of blue dye that was administered. We chose to inject the blue dye immediately after general anesthesia, in order to allow enough time for the blue dye to migrate through the lymphatics, and at the same time, to avoid the patients’ discomfort during injection. Regarding the blue dye quantity that was injected, we administered 1 mL in order to avoid possible adverse reactions. An improved rate of lymph node detection was reported by previous studies after the injection of 2–5 mL of blue dye [7,8]. Some studies discussed the fact that the surgeons’ experience in ARM technique might influence the detection rate [7,8,13]. Our study on ARM was the first in a center with no previous experience in the ARM procedure. Additionally, another important topic is that failing to identify the ARM lymph node in the anatomical region of the ALND is not necessarily a failed procedure; the lymphatic drainage of the arm might take place outside the limits of axillary dissection [9].

From our analysis regarding the possible factors that might influence the detection of ARM nodes, we showed that only the BMI is of statistical significance. The group of patients in which the identification of ARM nodes was successful had an average BMI of 25.06, as opposed to an average BMI of 30.12 in the group in which the detection was not possible. The possible influence of BMI on lymph node detection was discussed in previous studies, but a significant relationship has not yet been proven [7,8,13] The fact that a higher BMI may pose problems in the identification of ARM nodes is questioning the feasibility of the ARM technique in obese patients. We have analyzed a number of other factors in order to find the ones that might have an influence; some of them were discussed in other studies as well (age, tumor dimensions, neoadjuvant chemotherapy, lymph node status), while others were not previously mentioned (histological type, molecular subtype, histological grade) [7,14,15,16,17,18]. However, no statistically significant relationship was proven.

We found metastatic involvement of ARM nodes in 22.22%. This rate is higher than the one reported in a systematic review by Han et al.; metastases in ARM nodes were found in 16.9% (95% confidence interval—14.2–20.1%) [13]. In our cohort, when the axillary lymph node status was graded N2 or N3, ARM nodes were also involved. We were able to prove a statistically significant relationship between axillary lymph node status and ARM node metastases. This result is consistent with other studies [11,12]. Because of these outcomes, our study contradicts the initial premise on which the ARM procedure was built upon, that the lymphatic drainage of the arm and the lymphatic drainage of the breast take place on independent paths. We were not able to identify any preoperative factors that might predict the metastatic involvement of the ARM node, making it impossible to identify the patients who might be suitable candidates for the ARM technique. Considering the arguments above, we concluded that the oncological safety of axillary reverse mapping is questionable.

Most of the ARM nodes (96.3%) were identified in field A (above the intercostobrachial nerve, under the axillary vein and lateral to the thoracodorsal bundle), which proves there is a preferred anatomical lymph path for the drainage of the arm. Ikeda et al. also identified the most ARM nodes in field A, while Han et al. identified 58.76% of ARM nodes inferior to the axillary vein and lateral to the thoracodorsal bundle (field A, according to the Ikeda et al. classification) [2,19]. We analyzed the relationship between the anatomical localization and metastatic involvement of ARM lymph nodes, but we found no statistically significant relationship; since this is the first time this relationship is discussed, further analysis might be useful to be repeated on larger groups of patients.

Although our study was conducted on a small cohort, it has certain advantages compared to the other published studies: the ARM procedure was conducted by the same surgical team on all patients, with no variations regarding the technique; we analyzed a large number of variables; we found new statistical relations, not reported before and we proved for the first time the link between BMI and the detection rate of ARM nodes.

## 5. Conclusions

Axillary reverse mapping procedure, especially using blue dye, is an easy to reproduce technique in any surgical center, although the blue dye technique produces a relatively low detection rate. ARM node detection is difficult in patients with a high BMI, so the technique might not be feasible for these patients. There is a preferred anatomical pathway of drainage for the lymph of the arm (superior to the intercostobrachial nerve, inferior to the axillary vein and lateral to the thoracodorsal bundle). The rate of metastatic involvement of ARM nodes is significant and cannot be predicted by any preoperative factor; this shows that we cannot stratify breast cancer patients in order to identify the ones that might benefit from this procedure. However, we observed a correlation between ARM node metastases and the metastatic involvement of the other axillary lymph nodes, which might show that the drainage of the arm and breast have multiple connections that are not independent.

## Figures and Tables

**Table 1:** Population study and factors that might influence ARM node identification.

Parameter	Population Study	Identified ARM Nodes (*n* = 18)	Unidentified ARM Nodes (*n* = 12)	*p*-Value
Age *	56.73 ± 12.56	56.67 ± 12.61	56.83 ± 13.05	0.068
BMI *	27.08 ± 5.63	25.06 ± 4.78	30.12 ± 5.61	0.013
Tumor size **				0.35
T1	12	8	4	
T2	13	7	6	
T3	2	1	1	
T4	3	2	1	
Pathological lymph node status (pN) **				0.0001
pN0	14	10	4	
pN1	9	4	5	
pN2	5	2	3	
pN3	2	2	0	
Hystological type **				0.54
NST	25	15	10	
Lobular	4	3	1	
Other	1	0	1	
Intrinsic subtype **				0.39
Luminal A	16	5	11	
Luminl B Her 2−	13	7	6	
Luminal B Her 2+	6	4	2	
HER 2 overexpressed	1	1	0	
Triple negative	3	1	2	
Grade **				0.338
G1	4	2	2	
G2	18	12	6	
G3	8	4	4	
Neoadjuvant treatment **				0.51
ChT	15	7	8	
HT	4	2	2	
Localization **				0.52
UOQ	15	9	6	
UIQ	2	1	1	
LOQ	3	2	1	
LIQ	2	1	1	
Central	2	1	1	
Multicentric/multifocal	6	4	2	

* Variables described as average ± SD. ** Variables described as number of patients. BMI—body mass index. ChT—neoadjuvant chemotherapy. HT—neoadjuvant hormonal therapy. UOQ—upper outer quadrant. UIQ—upper inner quadrant. LOQ—lower outer quadrant. LIQ—lower inner quadrant.

**Table 2:** Factors that might influence metastatic involvement of ARM node.

Factor	Positive ARM Nodes	Negative ARM Nodes	*p*
Average age *	53.79 ± 10.05	66.75 ± 16.99	0.068
Average BMI *	24.67 ± 4.13	26.42 ± 7.23	0.53
Axillary lymph node status **			0.0001
pN0	10	0	
pN1	4	0	
pN2	0	2	
pN3	0	2	
Tumor size **			0.35
T1	7	1	
T2	5	2	
T3	1	0	
T4	1	1	
Histological grade **			0.338
G1	2	0	
G2	9	3	
G3	3	1	
Neoadjuvant chemotherapy **	6	1	0.51
Histological type **			0.54
NST	12	3	
Lobular	2	1	
Molecular subtype **			0.39
Triple negative	1	0	
Luminal A	4	1	
Luminal B Her2 positive	3	1	
Luminal B Her2 negative	6	1	
Her2 overexpression	0	1	

* Variables described as average ± SD. ** Variables described as number of patients. BMI—body mass index.

## Data Availability

The data presented in this study are available on request from the corresponding author. The data are not publicly available due to local regulations.
